# Multiparametric MRI for the improved diagnostic accuracy of Alzheimer’s disease and mild cognitive impairment: Research protocol of a case-control study design

**DOI:** 10.1371/journal.pone.0252883

**Published:** 2021-09-21

**Authors:** Albert Dayor Piersson, Buhari Ibrahim, Subapriya Suppiah, Mazlyfarina Mohamad, Hasyma Abu Hassan, Nur Farhayu Omar, Mohd Izuan Ibrahim, Ahmad Nazlim Yusoff, Normala Ibrahim, M. Iqbal Saripan, Rizah Mazzuin Razali

**Affiliations:** 1 Diagnostic Imaging and Radiotherapy Programme, Universiti Kebangsaan Malaysia, Kuala Lumpur, Malaysia; 2 Department of Imaging Technology & Sonography, School of Allied Health Sciences, University of Cape Coast, Cape Coast, Ghana; 3 Faculty of Medicine and Health Sciences, Department of Radiology, Universiti Putra Malaysia, Seri Kembangan, Malaysia; 4 Faculty of Medicine and Health Sciences, Neuroscience Laboratory for Cognitive Function and Behavioural Imaging (NeuroCoB), Universiti Putra Malaysia, Seri Kembangan, Malaysia; 5 Faculty of Basic Medical Sciences, Department of Physiology, Bauchi State University PMB 65, Gadau, Nigeria; 6 Faculty of Medicine and Health Sciences, Department of Psychiatry, Universiti Putra Malaysia, Seri Kembangan, Malaysia; 7 Faculty of Engineering, Department of Computer & Communication Systems, University Putra Malaysia, Seri Kembangan, Malaysia; 8 Gerontology Unit, Department of Medicine, Hospital Kuala Lumpur, Kuala Lumpur, Malaysia; University at Buffalo, UNITED STATES

## Abstract

**Background:**

Alzheimer’s disease (AD) is a major neurocognitive disorder identified by memory loss and a significant cognitive decline based on previous level of performance in one or more cognitive domains that interferes in the independence of everyday activities. The accuracy of imaging helps to identify the neuropathological features that differentiate AD from its common precursor, mild cognitive impairment (MCI). Identification of early signs will aid in risk stratification of disease and ensures proper management is instituted to reduce the morbidity and mortality associated with AD. Magnetic resonance imaging (MRI) using structural MRI (sMRI), functional MRI (fMRI), diffusion tensor imaging (DTI), and magnetic resonance spectroscopy (^1^H-MRS) performed alone is inadequate. Thus, the combination of multiparametric MRI is proposed to increase the accuracy of diagnosing MCI and AD when compared to elderly healthy controls.

**Methods:**

This protocol describes a non-interventional case control study. The AD and MCI patients and the healthy elderly controls will undergo multi-parametric MRI. The protocol consists of sMRI, fMRI, DTI, and single-voxel proton MRS sequences. An eco-planar imaging (EPI) will be used to perform resting-state fMRI sequence. The structural images will be analysed using Computational Anatomy Toolbox-12, functional images will be analysed using Statistical Parametric Mapping-12, DPABI (Data Processing & Analysis for Brain Imaging), and Conn software, while DTI and ^1^H-MRS will be analysed using the FSL (FMRIB’s Software Library) and Tarquin respectively. Correlation of the MRI results and the data acquired from the APOE genotyping, neuropsychological evaluations (i.e. Montreal Cognitive Assessment [MoCA], and Mini–Mental State Examination [MMSE] scores) will be performed. The imaging results will also be correlated with the sociodemographic factors. The diagnosis of AD and MCI will be standardized and based on the DSM-5 criteria and the neuropsychological scores.

**Discussion:**

The combination of sMRI, fMRI, DTI, and MRS sequences can provide information on the anatomical and functional changes in the brain such as regional grey matter volume atrophy, impaired functional connectivity among brain regions, and decreased metabolite levels specifically at the posterior cingulate cortex/precuneus. The combination of multiparametric MRI sequences can be used to stratify the management of MCI and AD patients. Accurate imaging can decide on the frequency of follow-up at memory clinics and select classifiers for machine learning that may aid in the disease identification and prognostication. Reliable and consistent quantification, using standardised protocols, are crucial to establish an optimal diagnostic capability in the early detection of Alzheimer’s disease.

## Introduction

Alzheimer’s disease (AD) and other dementias affects over 50 million people worldwide [1], about 30 million in Asia, and 123,000 people in Malaysia [2]. The latter is projected to be 261,000 by 2030 and will continue to increase to 590,000 people in 2050 [3]. Age is considered the most risk factor of AD as the incidence of the disease increases with the advancement of age [4]. The Malaysian population aged 60 years and over is projected to increase to about 7 million or 17.6% of the projected population of 40 million by 2040 [5]. In recent times, dementia and AD have become a major concern [6]. AD is a neurocognitive disorder identified by memory loss and a significant cognitive decline based on the previous level of performance in one or more cognitive domains that interfere in the independence of everyday activities.

It is characterized by neurodegeneration which initially affects the entorhinal cortex and then progresses to the hippocampus as a result of neuronal death that spreads with time to other parts of the brain [7]. The atrophy occurs due to the accumulation of toxic amyloid beta Aβ leading to the formation of plaques, neurofibrillary tangles and neuroinflammation [8] which may be triggered by genetic and environmental factors [9].

Fundamentally, AD may undergo a prodromal stage of mild cognitive impairment (MCI) before the appearance of pathological evidence of full-blown major neurocognitive deficit. MCI is defined by a progressive decline in memory, attention, judgement and executive function that is beyond what is expected of the individual’s age and level of education but does not interfere with daily life activities nor does it meet the criteria for dementia [10]. In a community-based study of Malaysians living in an urban area, it was estimated that about one-fifth of the older adults had MCI, with prevalence of 21.1% for all type of MCI, 15.4% for amnestic MCI, and 5.7% for non-amnestic MCI [11]. A longitudinal study (~1½ years follow-up) of older adults in Malaysia, also found that the male sex with less engagement in mental activities were predictors of developing MCI [12]. Nevertheless, health education with a focus on nutrition, lifestyle, and cognitive exercise has been shown to improve the nutritional status, knowledge, and attitude score of Malaysian older adults having MCI [13–15].

The neuropathology of AD has been studied using biochemical, genetics, neuroimaging, and histopathological biomarkers. Imaging biomarkers such as decreased fluorodeoxyglucose uptake on positron emission tomography/computed tomography (FDG-PET/CT), increased amyloid deposition detected by PET/CT amyloid imaging, and regional brain atrophy seen on structural high resolution magnetic resonance imaging (MRI) done separately have helped to improve the diagnostic accuracy of detecting AD [16,17]. The advantages of MRI are that it has an intrinsically high soft-tissue contrast resolution capability, does not utilize ionizing radiation, and is a potentially superior diagnostic tool to investigate the structural, functional, and neurochemical changes that occur in MCI and AD. The combination of multiple sequences such as structural MRI (sMRI), functional MRI (fMRI), diffusion tensor imaging (DTI), and proton magnetic resonance spectroscopy (^1^H-MRS), respectively are currently available for various neuroimaging purposes.

Conventionally, part of the routine clinical management of AD involves performing an sMRI examination to detect brain atrophy and to exclude other secondary causes of dementia. sMRI-detected brain atrophy that occurs in AD has a characteristic pattern, which involves the medial temporal lobes, paralimbic and temporoparietal cortices, and are considered as biomarkers of AD-related neurodegeneration [18]. However, gray matter (GM) volume is postulated to become abnormal only later in the course of the disease as a marker of neuronal loss [19], and it is not recommended as a stand-alone examination to diagnose early AD [20]. Various experimental studies have explored the incremental benefits of adding other structural MRI technique i.e. DTI and functional MRI sequences such as fMRI, and ^1^H-MRS [21]. fMRI is sensitive to measure and localize specific functions of the human brain, which may suffer from cognitive decline.

Resting state fMRI (rs-fMRI) is considered a promising technique for early detection of AD [22,23]. Rs-fMRI has been used to detect early AD by showing alteration of brain network connectivity such as default mode network (DMN) in the precuneus (Prec) and posterior cingulate cortex (PCC) and limbic networks [24]. DTI analyses have been widely used for AD studies and have successfully identified reduced fractional anisotropy (FA) or increased mean diffusivity (MD) within the splenium of the corpus callosum, the cingulum bundle, and the fornix [25]. Further, DTI parameters have been reported to provide a high cross-validated diagnostic accuracy of almost 80% for the clinical diagnosis of MCI and the discrimination of Aβ positive MCI cases from Aβ negative controls [26]. ^1^H-MRS measures brain chemistry is sensitive to neuronal changes. ^1^H-MRS metabolites i.e., N-acetyl aspartate (NAA), myo-inositol (mI), choline (Cho), creatine (Cr), NAA/Cr, NAA/ml, and mI/Cr ratios have been suggested as potential biomarkers of brain dysfunction in patients with AD [27–29]. However, to the best of our knowledge, no study has examined these imaging sequences in a multiparametric framework for interrogating the clinical stages of AD.

To this end, this study aims to investigate the combination of multimodal MRI in its ability to elicit structural, functional, and neurochemical changes in MCI and early AD compared to healthy elderly controls (HC).

### Study hypothesis

Multiparametric MRI, consisting of sMRI, fMRI, DTI and ^1^H-MRS performed in early AD and MCI compared with elderly HC is accurate to predict the risk of developing AD with neuropsychological tests and clinical diagnosis being the reference standard.

### Study objectives

The primary study objective is to investigate multiparametric MRI for risk assessment based on imaging biomarkers and prediction of AD. The imaging biomarkers will be correlated with sociodemographic factors and neuropsychiatric test scores. The clinical diagnosis of MCI and AD using on our institutional protocol based on the DSM-5 criteria will be utilised as a reference standard to classify the patients.

The specific objectives of the study, in order of importance, are:

1. to differentiate the structural, functional, and neurochemical changes among AD and MCI subjects compared to HC using multi-parametric MRI sequences
2. to determine the association between structural, functional, and neurochemical changes with sociodemographic factors, neuropsychological test scores, and clinical diagnosis of MCI and AD.
3. to evaluate the role of sMRI, fMRI, DTI, and ^1^H-MRS for risk prediction of developing early AD
4. to determine the association between socio-demographic risk factors and APOE genotyping in AD and MCI

## Materials and methods

### Study location

The study will be conducted in Hospital Kuala Lumpur and Universiti Putra Malaysia (UPM) in Malaysia.

### Ethical approval and consent to participate

We have obtained ethical approval from the Medical Research & Ethics Committee of the National Medical Research Register (NMRR-19-2719-49105) Malaysia, the Ethics Committee for Research Involving Human Subjects of UPM (JKEUPM-2019-328), and the Centre for Research and Instrumentation Management of Universiti Kebangsaan Malaysia (UKM PPI.800-1/1/5/JEP-2019-371) to carry out the proposed study. In this regard, all subjects (or Guardian/Legal Representative) will be issued an information sheet and required to provide written informed consent before participating in this study. The HC and MCI subjects will be psychologically evaluated by a clinician/ geriatrician. They will be able to personally give consent once they are declared fit to do so by the clinician. For the AD subjects, their next of kin, who is also authorised to decide on their treatment plan, will give consent on their behalf. For the subjects who may not be able to read, understand or give consent by themselves, their first-degree family member will be asked to consent on their behalf. This consent procedure has been duly approved by the Ethics Committee. Further, all information regarding the subjects’ participation in this research will be anonymous and the confidentiality will be maintained. All researchers in this research team will adhere to the principles of the Declaration of Helsinki and the Malaysian Good Clinical Practice Guidelines.

### Study design

This is a case-control study design. Patients and healthy elderly control subjects who confirm their participation will be requested to sign the informed consent form (or through the consent of their next-of-kin) and those who have agreed and signed the informed consent form will then meet geriatricians at the memory clinic in HKL to undergo neuropsychological examination (MoCA, MMSE, and CDR), which will involve an interview and completion of questionnaires. MoCA scores for HC, MCI, and dementia will be > 26, 24–26, and < 24 respectively; MMSE scores for HC, MCI, and dementia will be 24–30, 19–23, and < 19 respectively; and CDR scores for HC, MCI, and dementia will be 0, 1, and 2 respectively.

The subjects will also undergo PCR testing for APOE genotyping to correlate the presence of homozygous vs. heterozygous APOE genotype with the imaging biomarkers of AD and MCI.

For AD and MCI patients, neuropsychological testing will take place during their routine clinical appointment sessions at HKL memory clinic. In contrast, the first appointment will be booked for healthy control subjects to visit HKL for similar neuropsychological examination. In another separate occasion, all subjects will be required to visit UPM to undergo blood taking for APOE genotyping test, and a multiparametric MRI scan, of which the scan time is estimated to be 45 minutes long. The details of the study schematic, inclusion and exclusion criteria for both patients and control subjects are described below:

### Study schematic

**Fig 1:**
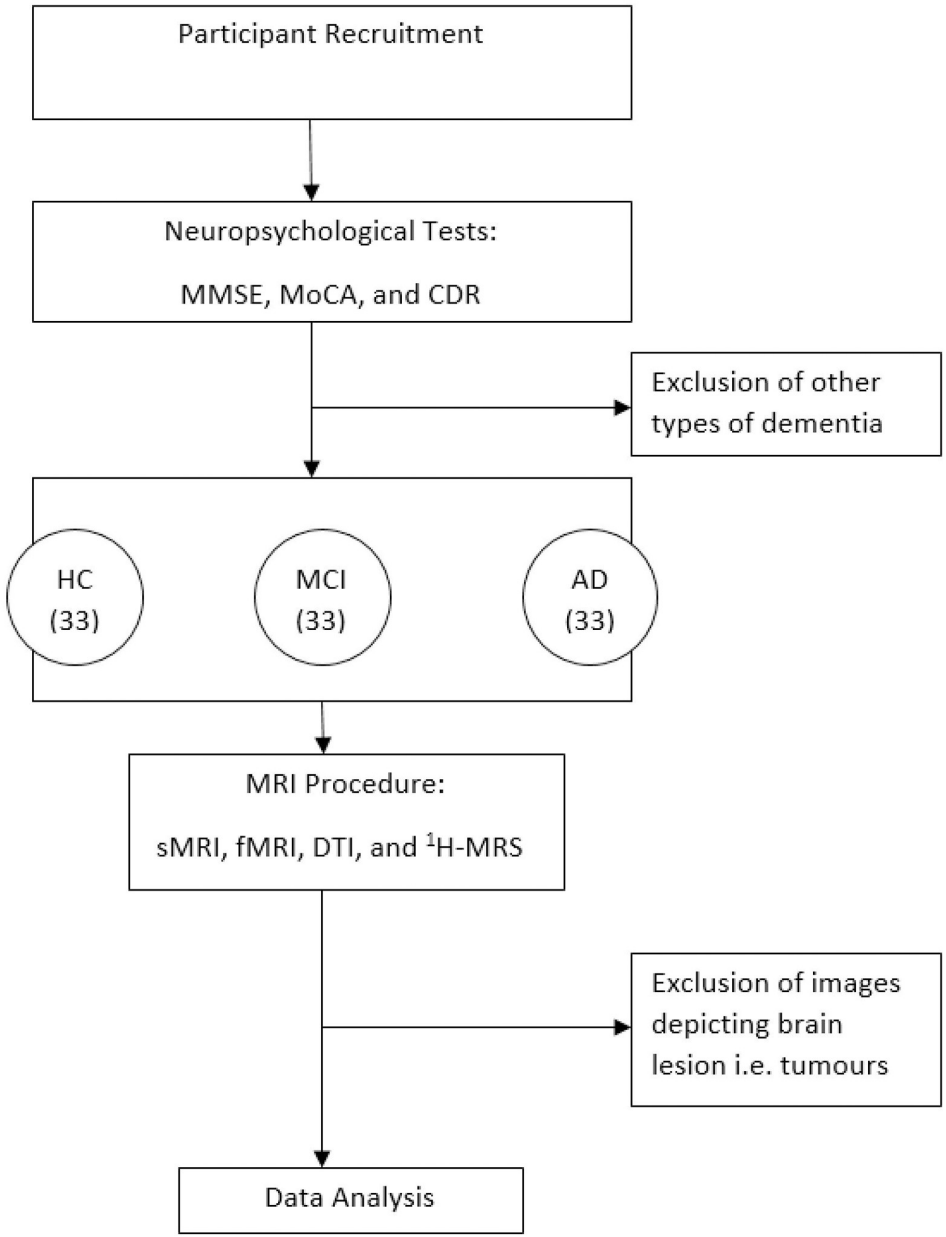
Methodology flowchart. MMSE = Mini-mental state examination, MoCA = Montreal Cognitive Assessment, CDR = Clinical dementia rating, HC = Healthy control, MCI = Mild cognitive impairment, AD = Alzheimer’s disease, sMRI = structural MRI, fMRI = functional MRI, DTI = diffusion tensor imaging, ^1^H-MRS = magnetic resonance spectroscopy.

All subjects will have a multiparametric MRI scan at one time point, within one month of the clinical assessment as shown in the methodology flowchart in Fig 1.

**Inclusion criteria for patients**
1. Malaysians
2. subjects aged 50–80 years old (males and females),
3. clinically confirmed diagnosis of MCI and AD by DSM-5 criteria
4. no other psychiatric, neurological, or cognitive impairment disorders, and
5. cooperative for MRI

**Inclusion criteria for healthy elderly controls**
1. Malaysians,
2. subjects aged 50–80 years old (males and females),
3. clinically not diagnosed to have AD by DSM-5 criteria or other no other psychiatric, neurological, or cognitive impairment disorders
4. cooperative for MRI

**Exclusion criteria for patient**
1. not within the expected age group,
2. foreigners,
3. diagnosis of AD is uncertain or presence of other no other psychiatric, neurological, or cognitive impairment disorders,
4. contraindicated for MRI study

**Exclusion criteria for healthy elderly controls**
1. not within the expected age group,
2. foreigners,
3. have neurological disorder or on medication(s) that might cause impaired cognitive function,
4. contraindicated for MRI study

### Sampling population

For study, the population of AD and MCI subjects will be recruited from patients attending the memory clinic of our institution to join the study, while their age-matched HCs will be recruited through advertisements in social and mass media in the in the regional districts surrounding the vicinity of the study site.

### Sample size

A total of 99 subjects (33 AD, 33 MCI and 33 HC) was calculated to be appropriate for the study. Initially, a sample size of a minimum of 25 subjects per group was calculated according to a similar study by with a power of 0.8 and an alpha significant level of 0.05 (two sided) [30] using G-power. Since subject drop-out is a common occurrence in this type of studies, the determined sample size can be inflated by 25% [31] making a final sample size of 33 subjects in each group. Thus, 33 AD subjects, 33 MCI and 33 HC will be recruited for multiparametric MRI scan.

### Study instrument and procedure

#### Magnetic resonance imaging

First, the procedure will be explained to all subjects. Each subject will undergo the multiparametric MRI separately according to their staggered appointment slots. On the day of their appointment, each subject will answer a checklist for MRI safety and be screened for any possible metal implants. All MRI scans will be acquired on a 3.0 Tesla Siemens Magnetom PRISMA (Model # 10849582; Serial # 66045, Magnetom, Erlangen, Germany) within the research institution’s MRI suite. A 64-channel phased-array head coil will be placed on the head of the subject. Prior to initiating the scan, the subjects will be instructed to keep their eyes closed (in the exception of the fMRI which will involve eye fixation), relax their minds and to avoid falling asleep, and not to move their heads. A foam pad and headphones will also be used to restrain head motion and scanner noise. Subsequently, the acquisition of sMRI, axial fluid attenuated inversion recovery (FLAIR), rs-fMRI, DTI, and ^1^H-MRS will be conducted.

#### Structural MRI

The structural MRI images will be acquired with a three-dimensional T1-weighted magnetization-prepared rapid gradient-echo (MPRAGE) sequence using the following parameters: TR (repetition time)/TE (echo time) = 2300/2.27 ms, inversion time = 900 ms, slice thickness = 1.0 mm, FoV (field of view) read = 250 mm, FoV phase = 100%, PAT (parallel acquisition technique) = 3, TA (acquisition time) = 3:54 minutes.

#### Coronal T2-weighted imaging

To evaluate for the hippocampal subfields, a coronal fast spin-echo T2-weighted images will be acquired using the following parameters: TR/TE = 4000/100 ms, slice thickness = 2.0 mm, in-plane resolution = 0.4 × 0.4 x 2 mm^3^ (slice plane to be angulated perpendicular to the long axis of the hippocampal formation), 30 interleaved slices, TA = 4:12 minutes.

#### Axial FLAIR imaging

To evaluate for white matter hyperintensities, whole-brain axial T2-weighted-FLAIR will be acquired using the following parameters: TR/TE = 4800/300 ms, slice thickness = 5.0 mm, distance factor = 25%, FoV read = 240 mm, FoV phase = 100%, PAT = 2, TA = 8:16 minutes.

#### Resting state fMRI (rs-fMRI) sequence

Whole-brain T2*-weighted (gradient echo) echo-planar images will be acquired at rest using the following parameters, in accordance with a previous study [32]: TR/TE = 2000/45 ms, slice thickness = 5.0 mm, distance factor = 25%, FoV read = 240 mm, FoV phase = 100%, PAT = 2, TA = 8:28 minutes

#### ^1^H-MRS sequence

A single-voxel in vivo ^1^H-MRS data will be obtained using a two-dimensional point-resolved spectroscopic pulse sequence (PRESS), as it is the most commonly used ^1^H-MRS technique for the study of AD [28]. The PRESS acquisition method offers the advantage of yielding 2-fold signal from its spin-echo compared to its counterpart, stimulated echo acquisition mode [33,34]. The following parameters will be used as recommended by a consensus group [33]: TR/TE = 2000/30 ms, flip angle = 90^0^, Time = 2.18 minutes. Although the TR value exerts a minimal effect on the spectra, it does affect the results by causing a varied scaling of the peaks [35,36]. Conversely, the TE is the most essential parameter, and a short TE of ~30 ms (compared to a longer TE) does not only permit the detection of more metabolites, but has an intrinsic signal-to-noise ratio (SNR) advantage compared to the use of long TE [35]. A 2 x 2 x 2 cm^3^ voxel will be placed in the midsagittal section of the PCC/Prec region. The ~ 8 cm^3^ voxel size is another commonly used 3-dimensional box that is recommended [34] as it can produce peaks with sufficient SNR. The PCC/Prec has been found to demonstrate cortical thinning [36], reduced glucose metabolism [37] and histopathologic changes [38] in the early course of AD. Further, previous large-scale ^1^H-MRS studies have selected the PCC/Prec for the placement of the ^1^H-MRS voxel [39,40] and, it was recommended by the MRS consensus group for ^1^H-MRS studies in AD [41]. An inversion pulse sequence is then applied to suppress the lipid signal outside the ~ 8 cm3 voxel size to avoid contamination with signal emanating from the metabolites.

#### Diffusion imaging

The following diffusion imaging parameters will be used, modified from previous studies [42,43]: TR/TE = 3400/71 ms, slice thickness = 4.0 mm, echo spacing = 0.6 ms, distance factor = 30%, diffusion directions = 20, Time = 4.17 minutes. Therefore, for each scan, the EPI acquisitions will include non-diffusion-weighted images (b = 0 s/mm^2^), mean diffusion (b = 1000 s/mm^2^), mean apparent diffusion coefficient (ADC) images, and colour fractional anisotropy images.

### MRI data pre-processing analysis using SPM 12

#### Structural data

**Fig 2:**
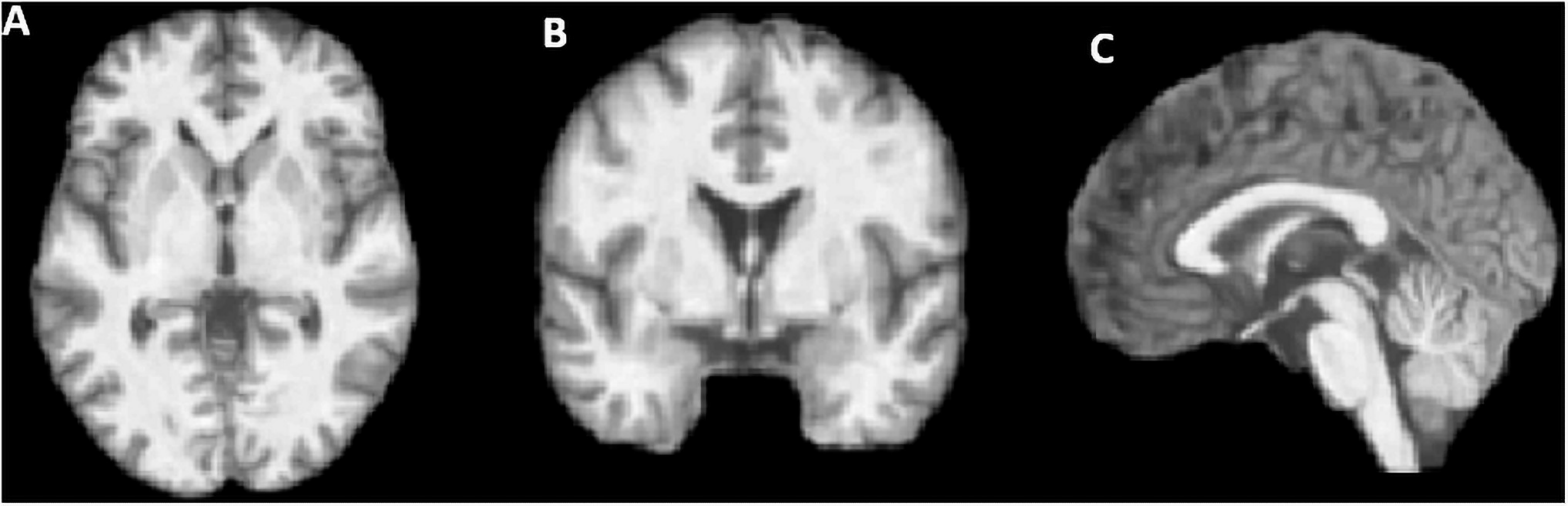
T_1_-weighted skull-stripped images of a healthy volunteer in the axial (A), coronal (B), and sagittal (C) planes of a volunteer.

The structural images will be pre-processed using the CAT12 toolbox (http://www.neuro.uni-jena.de/cat/) which runs within the Statistical Parametric Mapping 12 package (SPM12) (http://www.fil.ion.ucl.ac.uk/spm/software/spm12/; The Welcome Trust Centre for Neuroimaging, London, UK) using MATLAB 2019a. Briefly, the following steps will be used: normalisation of T1 images to a template space and segmentation of images into GM, white matter, and cerebrospinal fluid; visual inspection of images; estimation of total intracranial volume; inspection of data homogeneity; smoothing (using a full-width at half-maximum 8 mm Gaussian kernels) (Fig 2). GM volume will be used to assess whole-brain volumetric differences within and between groups. Threshold will be set at p < 0.05 (family-wise error, with correction for multiple comparisons) to investigate voxel-wise differences among groups.

#### Functional data

**Fig 3:**
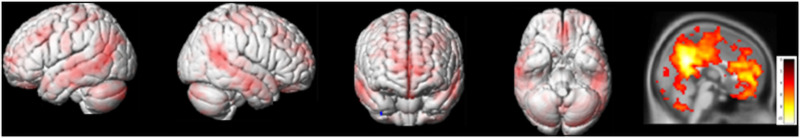
rs-fMRI of a healthy volunteer. Three-dimensional MNI surface renders and mean T_1_ weighted image of rs-fMRI.

The functional images will be pre-processed using SPM12 with the following steps: slice-timing correction; spatial realignment; co-registration to the T_1_-weighted anatomical image; spatial normalization to a standard template (Montreal Neurological Institute template, MNI); smoothing (using a 6mm FWHM Gaussian kernel) (Fig 3). Threshold will be set at p < 0.05 (family-wise error (FWE), with correction for multiple comparisons) to investigate voxel-wise differences among groups. Functional connectivity analyses will also be undertaken using DPABI (Data Processing & Analysis for Brain Imaging) [44] and the Conn toolboxes [45]. A total of 22 regions-of-interest (ROI) corresponding to the nodes of the default mode network (DMN), dorsal attention network (DAN), frontoparietal network (FPN), and salience network (SN) will be identified from the “networks atlas” and implemented in the Conn toolbox for the functional connectivity analyses [46].

#### ^1^H-MRS data

**Fig 4:**
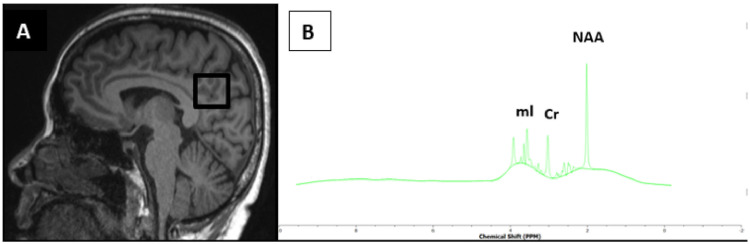
A midsagittal T_1_-weighted image of the brain of a healthy volunteer. A 2 x 2 x 2 cm^3^ voxel placement in the PCC/Prec is shown in *A*. The proton spectra obtained from the region with a TE of 30 ms depicts the NAA, Cr, and mI metabolites in *B*.

The ^1^H-MRS data will be processed in the Tarquin software [47] which involves the following pre-processing steps: removal of residual water signal, performing a preliminary phase estimation, calibration of the part per million axis based on spectral features, and fitting the data. A sample data is displayed in Fig 4 obtained from a healthy volunteer.

#### DTI data

**Fig 5:**
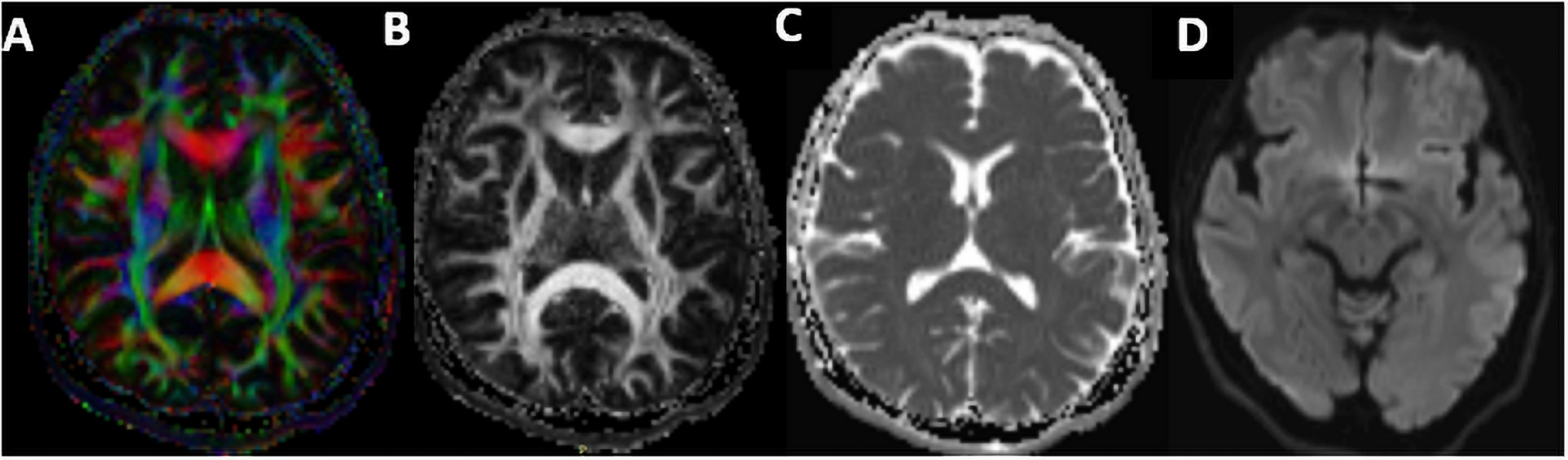
Axial DTI maps of colour FA (*A*), non-colour FA (*B*), ADC (*C*), and trace-weighted (*D*).

The DTI data (Fig 5) will be pre-processed using the FSL tool library (http://www.fmrib.ox.ac.uk/fsl) and the following steps: correction for eddy current distortion and head motion through affine registration of diffusion-weighted images to the non-diffusion weighted images (b = 0 s/mm^2^); rotation will be applied to the diffusion gradients so as to improve consistency with the motion parameters; FA, MD, radial diffusivity (RD), and axial diffusivity (DA) images will be created by fitting the diffusion tensor model to the diffusion-based data at each voxel level. Furthermore, tract-based spatial statistics [48] will be used to perform a whole-brain voxel-wise analysis with a focus on major white matter pathways. First, non-linear registration of the FA images to a high-resolution FA image will be undertaken and will further be skeletonised.

For the analysis of the structural and fMRI images, SPM12 will be used to compute 1-sample t-tests in a General Linear Model framework on each corresponding map and for each of the groups to determine the differences within the groups. Furthermore, the Dunnett-t test will be computed to determine the differences among the case group relative to the control group. For the structural data, the following covariates of no interest will be specified in the GLM–age, sex, and total intracranial volume. For both tests, the statistical threshold will be set at p <0.005 (family-wise error, correction to be performed for multiple comparisons). Activation clusters will be considered statistically significant if they are ≥ 10 voxels and attain an uncorrected voxel-wise significance level of p < 0.001 and a cluster whole-brain FWE corrected level of p < 0.05 [49]. These thresholds will then be used for the linear regression models in the main analysis of the whole study.

#### Multiparametric imaging analysis

A radiologist with 5 years’ experience in dedicated neuroimaging (SS), and a medical physicist with 10 years of experience in neurology image processing (MFM) will supervise the PhD candidates (ADP & BI) to perform image segmentation.

The ROI for analysis will be defined in the areas defined by *a priori* knowledge.

### Statistical analysis

A target of 99 subjects (33 AD, 33 MCI and 33 elderly HC) will be recruited and this is estimated to take 10 months. For estimated accuracy of detecting early AD using multiparametric biomarkers of 25%, 99 patients will provide 80% power to detect an area under curve ≥ 80%. Poor correctness of fit is estimated to be in the range of 25–45%.

SPSS version 23.0 will be used for the analysis. Descriptive analysis will be used for the categorical data and the numerical clinical data will be presented as the means and standard deviations. Data would be checked for outliers and normal distribution, as assessed by boxplot and Shapiro-Wilk test. Chi-square will be used to determine the association between AD and MCI in terms of their socio-demographic risk factors while independent t-test will be used to determine the differences between the sexes. Descriptive analysis will be used to display the neuropsychological test scores and imaging results of the subjects. T-test will be used to find the association between the neuropsychological test scores with each imaging classifier. One-way ANOVA will be used to correlate the various imaging classifiers among the 3 categories of AD, MCI, and HC. Multinomial logistic regression will be used to determine the association between structural, functional, and neurochemical changes with neuropsychological test scores in AD, MCI, and HC. A threshold of p < 0.05 will be considered statistically significant. However, where the normal Gaussian distribution of the data is not met, an equivalent non-parametric test will be applied. Upon determination of the most sensitive classifier or parameters, we will generate a receiver operating characteristic curve to achieve the specificity and sensitivity of the parameters to discriminate each patient group from the healthy control group and prediction of the diagnosis of AD. Furthermore, to have a robust prediction collinearity of the variables would be evaluated.

There are good prospects for applying deep learning, by identifying major variables to be used as classifiers, e.g. hippocampal and other cortical and subcortical GM volumes, and measures from fMRI, DTI, and ^1^H-MRS indices. Furthermore, a longitudinal study will be designed in the future to look for the MRI measures of interest that can aid in the prediction of risk and potential MCI to AD converters. Linear support vector machine classifiers and permutation tests can be utilised to identify which model of combined imaging biomarkers and clinical data can provide better diagnostic accuracy. Subsequently, the data will be copyrighted and will be made available to researchers upon reasonable request to the corresponding author.

## Discussion

### Primary outcome measures

1. Differences in neuropsychological scores among healthy controls, MCI, and AD patients will be evaluated. These measures include MMSE, MoCA, and CDR.
2. Differences in macro- and micro-structural changes, rs-fMRI, and ^1^H-MRS metabolites among healthy controls, MCI, and AD patients will be evaluated. These MRI measures will include volumetric characteristics (i.e. regional and whole-brain atrophy), functional characteristics (i.e. activated regions), microstructural tissue properties (i.e. FA, MD, RD, and DA), network properties (e.g., functional and structural connectivity), and H-MRS metabolite characteristics (NAA, mI, Cho, NAA/Cr, NAA/mI, and mI/Cr) correlated to APOE genotyping and neuropsychological test scores.

## Conclusion

### Implications of this protocol

Despite the extensive research conducted in this area, there is the pressing need for the development of neuroimaging biomarkers for early detection of risk of AD in normal cognitive aging, as well as prediction and monitoring of disease progression, and evaluation of treatment efficacy. Structural, functional, and neurochemical changes in brain regions have each been implicated in MCI and AD. However, there are no studies that have examined these imaging modalities in a multimodal framework.

This study therefore aims to investigate the combination of multiparametric MRI in their ability to elicit the best combination of neuroimaging markers for early diagnosis of MCI and AD before clinical symptoms becomes apparent. In addition, the study aims to apply risk stratification for the detection of possible indicators to identify and predict converters from MCI to AD. This study offers a rare and unique opportunity different from other studies because it considers the combination of multimodal MR imaging techniques in a single examination that can provide in-depth details of the whole brain. The limitation of this study is that it is a case-control study design that evaluates the subjects at a single time point. Hence the need to design a longitudinal study in future.

In conclusion, we have developed a protocol for a multiparametric neuroimaging model focused on early detection of MCI and AD. We anticipate that this model may serve as a diagnostic tool for the assessment of the AD continuum, and further our understanding regarding the mechanisms underlying the pathophysiological process of AD.

### Data privacy and confidentiality

All subjects’ medical information will be kept confidential and will not be made publicly available unless disclosure is required by law. Their samples/Data will be anonymous (non-identifiable) (i.e., personal identifiers will not be kept with the subject’s sample/Data and the sample/Data will not have a code number that can be used to identify the subject) or coded and considered de-identified (i.e., any identifying information such as name will be replaced with a code and only a few authorized people will have access to this code to link samples and data back to personal identifiers). Subjects may be allowed to have access to their personal data if they request for it.

## Supporting information

S1 Data Healthy volunteer MRI DICOM data (raw) acquired at 3.0 T. (ZIP)
